# Quantifying the Forces Applied During Manually and Mechanically Assisted Calvings in Beef Cattle

**DOI:** 10.3389/fvets.2020.00459

**Published:** 2020-08-05

**Authors:** Jennifer M. Pearson, Charmaine Thomsen, Ann Kusler, Edmund A. Pajor, Akshay Gurdita, Mark David Ungrin, M. Claire Windeyer

**Affiliations:** ^1^Department of Production Animal Health, University of Calgary Faculty of Veterinary Medicine, Calgary, AB, Canada; ^2^Department of Mathematics and Statistics, University of Calgary Faculty of Science, Calgary, AB, Canada; ^3^School of Optometry and Vision Science, University of Waterloo, Waterloo, ON, Canada; ^4^Department of Comparative Biology and Experimental Medicine, University of Calgary Faculty of Veterinary Medicine, Calgary, AB, Canada

**Keywords:** beef, calving assistance, dystocia, calving duration, force measuring device, calf jack

## Abstract

Dystocia is a leading cause of calf mortality, yet there is little available information quantifying the duration and forces applied to assisted deliveries. Objectives of this study were to: (1) develop a method to measure the magnitude and duration of various forces applied to a calf during calving assistance, and (2) quantify the forces applied to beef calves during manual or mechanical calving assistance. Twenty-five primiparous dams requiring calving assistance were enrolled. Calvings were assisted by manual (1 or 2 people pulling) or mechanical (calf extractor) delivery. A set of modified obstetric chains with integrated force measuring devices (Calving Assistance Force Logger; CAF-Log) were applied to the calf for delivery. The CAF-Log system was calibrated using known masses ranging from 25 to 200 kg in increasing increments of 25 kg. Duration of the assisted delivery and force parameters (peak force applied to one leg, peak force applied to both legs, cumulative force, and maximum jerk force) were described and assessed for their associations with method of delivery and ranch. Median duration was 112.6 s (IQR: 88.4–149.7) for manual and 312.6 s (IQR: 221.6–462.3) for mechanical deliveries. Mean peak force applied to one leg was 56.9 kg (SD: 22.9) for manual and 126.8 kg (SD: 48.2) for mechanical deliveries. Mean peak force applied to both legs was 95.4 kg (SD: 34.1) for manual and 188.6 kg (SD: 83.9) for mechanical deliveries. Median cumulative force was 178.3 kg min (IQR: 21.1–38.8) for manual and 380.6 kg min (IQR: 252.1–581.3) for mechanical deliveries. The maximum jerk force for manual deliveries was 36.6 kg/s (IQR: 21.1–38.8) and 77.2 kg/s (IQR: 60.9–97.1) for mechanical deliveries. An interaction occurred between ranch and method of delivery for peak force applied to one leg, peak force applied to both legs, and cumulative force. The CAF-Log system demonstrated that significantly greater forces were applied to mechanically delivered calves compared to manually delivered calves and could be used in future studies to investigate forces applied to a calf during calving assistance and their impacts on cow and calf well-being.

## Introduction

Calving difficulty negatively impacts overall herd productivity due to increased calf morbidity and mortality (1–3), reduced subsequent fertility and production in cows (4–6), and increased labor inputs by ranch personnel (7). Severe calving difficulty often results in a compromised calf (8). Specifically, the incidence of fetal trauma (9–11), broken bones and dislocated joints (12), and hypoxia and mixed respiratory and metabolic acidosis (13–16) increase with the severity of calving difficulty, and these directly impacts the vigor, transfer of passive immunity, health, and performance of the calf (11, 16–18).

Calving assistance is performed regularly by both cattle producers and veterinarians when failure of progression is observed during parturition (3, 19, 20). The incidence of assisted calvings in western Canada ranges from 5 to 9% (3, 20) with feto-pelvic disproportion being the leading cause of calving assistance in beef cattle (5, 21). High birthweight of the calf is a good predictor of calving difficulty (5, 21, 22). Calving difficulty is a subjective measure used to describe the amount of effort or force required to extract the calf (5). Reports quantifying the forces applied to extract a calf are limited in the literature to either newborn calf cadaver models or only one or two types of forces measured (23–25). A device that can digitally measure various types of forces applied during live calving assistance has not been described and may be useful to objectively classify calving difficulty when assessing the impacts of calving difficulty on cow and calf health. Therefore, the objectives of this study were to: (1) develop a method to measure the magnitude and duration of various forces applied to a calf during calving assistance, and (2) quantify the forces applied to beef calves during manual or mechanical calving assistance.

## Materials and Methods

### Development of the Calf Assistance Force Logger (CAF-Log)

**Figure 1:**
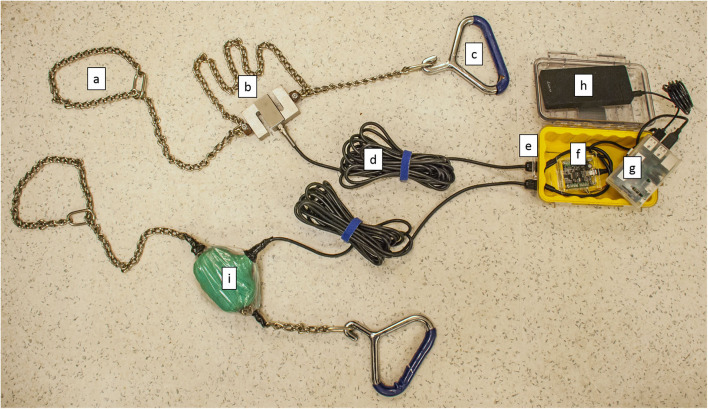
Modified obstetrical chains containing force measuring devices (Calf Assistance Force Logger; CAF-Log) used to measure the force applied during assisted deliveries of calves. **(a)** End of modified obstetrical chain looped around the leg of the calf. **(b)** S-type load cell (Chain 1). **(c)** Calving handle at the end of the modified obstetrical chain where force was applied. **(d)** Cable connecting load cell to analog-to-digital converter. **(e)** Modified waterproof, crush-proof case. **(f)** Analog-to-digital converter. **(g)** Single board computer. **(h)** Portable power supply. **(i)** S-type load cell (Chain 2) wrapped in bandaging materials and a plastic bag secured with electrical tape for field use.

Obstetrical chains were modified to measure the amount of force placed on manually (one or two people pulling to deliver the calf) and mechanically (fetal extractor, i.e., calf jack) delivered calves. The CAF-Log system consisted of several components (Figure 1). A S-type load cell (3140_0, Phidgets Inc, Alberta, Canada) was attached to the middle of an obstetrical chain in two places. The load cell was connected via a high-resolution analog-to-digital converter (ADC -PhidgetBridge 4-Input 1046_0, Phidgets Inc, Alberta, Canada) to a Raspberry Pi (RPi) single board computer (Raspberry Pi Foundation, UK) and run by a Raspbian (Debian, version 8) operating system. Custom Python software (Python Software Foundation, Oregon, USA) was activated automatically when the RPi was plugged into a portable power supply (Luxa2, EnerG Slim 10,000 mAh Power Bank, Thermaltake Technology Co., China). The script repeatedly read and recorded raw load cell data in voltage (mV/V) from the load cell every 0.2 s and recorded timestamped data to a Universal Serial Bus (USB) drive for later analysis. The ADC, RPi, and power supply were housed in a modified waterproof, crush-proof container (Pelican 1060 Micro Case Series, Pelican Products Torrance, CA, USA), with wires leading to the load cells (Figure 1).

### Instrument Calibration

To convert raw ADC data recorded by the CAF-Log system into force parameters, each chain of the CAF-Log was independently calibrated by suspending known masses (ranging from approximately 25 to 200 kg in increasing increments of 25 kg) using a hydraulic lift for 3 min to ensure an extensive period of stable readings. The output of the device when lifting known masses was used to calculate a conversion factor (K; see section Force Calculations).

### Field Application

The field study was conducted in accordance with the guidelines established by the Canadian Council on Animal Care with approval granted on January 5, 2016 by the University of Calgary Veterinary Sciences Animal Care Committee (AC15-0150). Data were collected during the 2016 calving season (January to May) from two ranches in southern Alberta, Canada from cow-calf pairs that were enrolled as previously described (26). Twenty-five heifers (Ranch A = 16; Ranch B = 9) assisted in delivery of their calf using the modified obstetrical chains were enrolled. Twins and deliveries by caesarian section were not enrolled in this study. Pregnant dams were monitored for signs of parturition hourly in outdoor pre-calving pens close to the calving barns. Dams were moved to a calving chute for vaginal examination and assisted delivery of the calf if they failed to calve or make progression within one to 2 h of estimated onset of stage two labor (e.g., amniotic sac visible, feet present, strong abdominal contractions, etc.) as determined by the ranch personnel. The definition of stage two labor begins with dilation of the cervix and ends with expulsion of the fetus (27). The onset of stage two by this definition cannot be determined without vaginal examination so the visually estimated onset of stage two labor described above was used for this study. If assistance was required, the modified obstetrical chains were placed on the legs of the calf using the double half hitch method (7): the proximal loop was placed at the narrowest point of the metacarpus and the distal loop was placed between the dewclaws and the hoof. The chains were then attached to either obstetrical hooks for manual delivery (Figure 1) or a fetal extractor (Dr. Frank's Calf Puller, Ideal Instruments Inc.) for mechanical delivery, as determined by the discretion of ranch personnel. Forces were recorded and uploaded to the USB drive for later analysis. Within 10 min after calving assistance, the date and time of calving, method of delivery (manual or mechanical), and calf birthweight using a digital scale were recorded. Calves were evaluated at birth for obvious signs of injury (e.g., fractures, swelling) and calves that died during the preweaning period were submitted to the University of Calgary Faculty of Veterinary Medicine Diagnostic Services Unit for gross and histological examination to determine the cause of death.

### Force Calculations

The K factor and measured forces were calculated using the statistical software R (Version 3.3.2, R Foundation for Statistical Computing, Vienna, Austria; www.r-project.org). The K factor was calculated by plotting the voltage (mV/V) of the force measuring device's output by the known increasing mass increments from the instrument calibration (section Instrument Calibration). A linear regression model was used to analyze the relationship between the measured force and the known masses, and the inverse of the slope was the calculated K factor for each chain. To determine whether there was a difference in the K factors between the two chains, an Analysis of Covariance test was performed (28). Chain 1 and chain 2 were described as categorical variables and two regression models were created: one modeling the interaction between the categorical variable and the voltage, and one without the interaction. These models were then compared using an ANOVA.

To determine the expected force applied during each assisted delivery in kg, the calculated K factors for each chain were applied to the device output from the field study using the following equation:

$$\begin{array}{l} \text{F}=\text{K}\times \left(\text{m}-\text{o}\right) \end{array}$$

Where F = expected force, K = proportionality constant calculated by calibrating each chain, m = the measured voltage, and o = offset. The measured voltage was the voltage output of the device for each chain in mV/V, and the offset was the baseline voltage in mV/V. The baseline voltage was determined by calculating the average voltage measured by each chain at 2,000 or more timepoints, with no load on the system, after a calf was delivered. It is important to note that although kg is not a force unit, the above equation calculates a value equivalent to measuring the force due to gravity. Therefore, in this study the unit of kg was used to describe forces placed on the calf for ease of interpretation. The measurement of Newtons can be converted to kilograms (kg) by dividing Newtons by the acceleration of gravity on earth (9.81 m/s).

After converting the voltage output data into force using the above equation, the output was plotted graphically for each chain and for each calving. Duration (sec) was defined as the interval between the timepoint when the force of the first chain moved above baseline until the timepoint when the last chain's force dropped below that threshold for the last time based on visual assessment. Peak force (kg) was calculated as the highest measured force value applied at any single timepoint during the assisted delivery for each chain. To calculate the peak force applied to one leg (kg), the maximum force at a single timepoint for either chain 1 or chain 2 was determined. To calculate the peak force applied to both legs (kg), the forces for chain 1 and chain 2 at each timepoint were summed and the maximum force value was determined. The cumulative force (kg min) was calculated by determining the change in force (subtracting the force at the previous timepoint from the force at each timepoint) and summing all positive changes of force for the duration of the assisted delivery. The maximum jerk force was calculated using the peak change in force, with all negative changes in force set to zero, divided by 0.201 s (time interval between timepoints). The jerk force value was calculated by averaging the mean of the jerk force at a single timepoint and the jerk force at the two prior timepoints and two subsequent timepoints to create a force value for one second. The maximum jerk force (kg/sec) was the highest of these values that occurred over the duration of the assisted delivery.

### Statistical Analysis

Data were analyzed using STATA® 14.1 software (StataCorp LP, College Station, TX) to investigate the relationship of the duration and forces applied at delivery with method of delivery (manual or mechanical) on two ranches (Ranch A and Ranch B). Descriptive statistics and tests for normality were performed on all continuous variables. To compare the proportions of calves assisted with each method of delivery by ranch and sex of the calf, a Fisher's Exact test was performed. To compare birthweights by method of delivery and ranch, a Wilcoxon Rank Sum test was performed for this non-parametric variable. Multivariable linear regression models were used to evaluate associations between method of delivery and outcome forces (duration, peak force applied on one leg, peak force applied on both legs, cumulative force, and maximum jerk), while accounting for ranch. Multicollinearity was assessed using Spearman's rank correlation. Univariable analysis was performed on all covariates (method of delivery and ranch) using a *P* ≤ 0.15 as the inclusion criterion for the models (29). All models were analyzed using a forward selection model building strategy (29). The significance level for variables to be retained in the model was set at α = 0.05. Regression models were checked for assumptions by Cook-Weisberg test for heteroscedasticity and Shapiro-Wilk W test for normality. Residuals were assessed visually by residual-versus-fitted plots. All significant covariates were checked for interactions within each model.

## Results

### Instrument Calibration

The K factor value for chain 1 was 0.00400 and for chain 2 was 0.00395 (*P* = 0.03). Due to this statistical difference between K factors for chain 1 and chain 2, separate K values for each chain were used when calculating forces for the field application.

### Field Application

Of the 25 enrolled assisted calvings, 7 (28%) were delivered by manual extraction and 18 (72%) were delivered by mechanical extraction. Three of the 25 calves died due to dystocia-related causes (manual = 0; mechanical = 3). One calf died at 4 h of age due to hypoxia and acidemia associated with a prolonged birth, one calf died at 48 h of age due to meconium aspiration, and one calf died at 4 days of age due to omphalitis and meconium aspiration. No fractures or other major signs of injury (e.g., bruising, swelling) were identified in calves enrolled. Sixteen calves were enrolled on Ranch A and 9 calves on Ranch B. Of the calves enrolled on Ranch A, 4 were manually delivered and 12 were mechanically delivered. Of the calves enrolled on Ranch B, 3 were manually delivered and 6 were mechanically delivered. There was no difference in the proportion of calves delivered by each method of delivery between Ranch A and Ranch B (P = 0.7). Calves had a lower median birthweight on Ranch A (37.3 kg, IQR: 33.1–41.3) compared to Ranch B (48.2 kg, IQR: 40.5–50.0) (P = 0.004). When comparing manual (37.5kg, IQR: 30.9–39.3) and mechanically (40.9 kg, IQR: 36.5–46.6) delivered calves' birthweight, there was not a significant difference overall (P = 0.1). However, the birthweights by ranch were significantly different between mechanically delivered calves on Ranch A (39.3 kg, IQR: 34.6–42.8) and Ranch B (49.6 kg, IQR: 41.4–50.8) (P = 0.003), but no significant difference between manually delivered calves' birthweight on Ranch A (34.0 kg, IQR: 27.1–37.3) and Ranch B (39.3 kg, IQR: 37.7–48.2) (P = 0.1). Of the 25 calves enrolled, 7 were heifers (manual = 3, mechanical = 4) and 18 were bull calves (manual = 4, mechanical = 14). There was no difference in the proportion of heifer and bull calves delivered by each method of delivery (P = 0.3), so calf sex was not considered as a potential covariate in the multivariable models.

**Figure 2:**
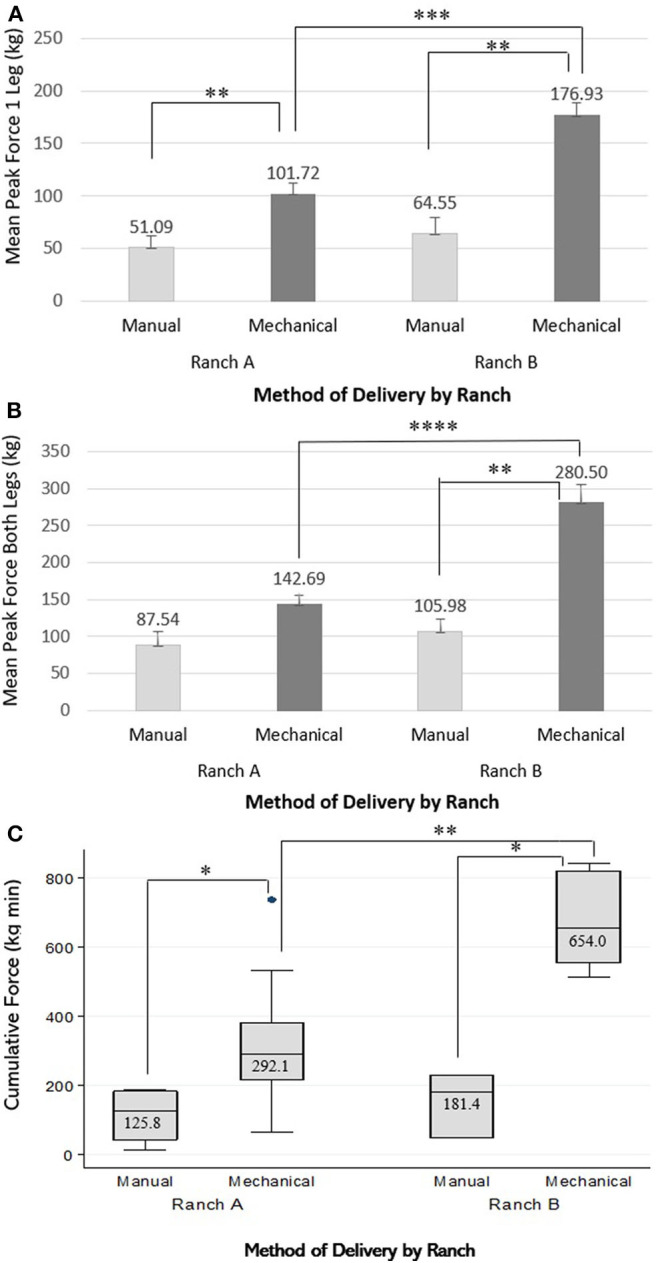
Visual description of the effect of an interaction between method of delivery (Manual or Mechanical) and ranch (Ranch A or Ranch B) on **(A)** mean peak force on one leg (kg), **(B)** mean peak force on both legs (kg), and **(C)** cumulative force (kg min) for assisted delivery of 25 beef calves on 2 ranches. *P ≤ 0.05, **P ≤ 0.01, ***P ≤ 0.001, ****P ≤ 0.0001. The number of calves manually delivered on Ranch A was 4 and on Ranch B was 3, and the number of calves mechanically delivered on Ranch A was 12 and on Ranch B was 6.

The distribution of force parameters and comparison by the method of delivery are described in Table 1. Duration and all forces were greater for mechanically extracted calves than manually extracted calves during univariable analysis (*P* < 0.05). Table 2 describes the associations between the predictors (method of delivery and ranch) for the different measured forces. For peak force applied on one leg, peak force applied on both legs, and cumulative force, there was a ranch by method of delivery interaction (Figure 2), whereby forces were greater for mechanically delivered calves on Ranch B compared to manually delivered calves on Ranch B (*P* < 0.05). Peak force applied on one leg and cumulative force were greater for mechanically delivered calves on Ranch A compared to manually delivered calves on Ranch A (*P* < 0.05). Forces were greater for mechanically delivered calves on Ranch B compared to mechanically delivered calves on Ranch A (*P* < 0.05), but there were no differences in forces between manually delivered calves on Ranch A and Ranch B (*P* > 0.05).

**Table 1:** Description and comparison of the duration and various forces applied for manual or mechanical calving assistance of 25 beef calves on 2 ranches.

	**Manual (*****n*** **=** **7)**			**Mechanical (*****n*** **=** **18)**			***P* value**
	**Mean/** **median**	**Standard deviation/** **interquartile range**	**Range**	**Mean/** **median**	**Standard deviation/** **interquartile range**	**Range**	
Duration (s)^*^	112.6	88.4–149.7	19.1–168.8	312.6	221.1–462.3	116.6–635.2	0.0003
Peak force applied on one leg (kg)	56.9	22.9	18.6–94.8	126.8	48.2	43.1–213.9	0.001
Peak force applied to both legs (kg)	95.4	34.1	28.6–139.6	188.6	83.9	63.8–346.2	0.009
Cumulative Force (kg min)^*^	178.3	47.9–186.8	12.1–230.1	380.6	252.1–581.3	63.9–842.6	0.001
Maximum Jerk (kg/s)^*^	36.6	21.1–38.8	11.7–76.3	77.2	60.9–97.1	34.2–112.2	0.002

* *Values described are median and interquartile range*.

**Table 2:** Significant predictors of duration and various forces applied for calving assistance of 25 beef calves on 2 ranches as determined by multivariable linear regression modeling.

	**Coefficient**	**Standard error**	***P* value**
**DURATION (S)**			
Method of delivery			
Manual	Referent	–	–
Mechanical	230.6	59.6	0.001
**PEAK FORCE APPLIED TO ONE LEG (KG)**			
Method of Delivery			
Manual	Referent	–	–
Mechanical	50.6	17.7	0.009
Ranch			
Ranch A	Referent	–	–
Ranch B	13.5	23.4	0.6
Ranch by method of delivery interaction^*^	61.8	27.9	0.04
**PEAK FORCE APPLIED TO BOTH LEGS (KG)**			
Method of delivery			
Manual	Referent	–	–
Mechanical	55.2	28.2	0.06
Ranch			
Ranch A	Referent	–	–
Ranch B	18.4	37.3	0.6
Ranch by method of delivery interaction^*^	119.4	44.5	0.01
**CUMULATIVE FORCE (KG MIN)**			
Method of delivery			
Manual	Referent	–	–
Mechanical	209.9	87.7	0.03
Ranch			
Ranch A	Referent	–	–
Ranch B	40.5	115.9	0.7
Ranch by method of delivery interaction^*^	309.7	138.6	0.04
**MAXIMUM JERK (KG/S)**			
Method of delivery			
Manual	Referent	–	–
Mechanical	38.9	9.4	<0.0005

* *Interaction terms described within the text and Figure 2*.

## Discussion

The CAF-Log system developed for this study successfully enabled measurement of various force parameters applied to calves during both manual and mechanical assisted deliveries under field conditions. In general, longer durations, greater peak forces, greater cumulative force, and greater maximum jerk force were associated with mechanical deliveries compared to manual deliveries, although this did sometimes vary by ranch.

Similar to the forces applied during deliveries in the present study, it has been reported that the applied force exerted by 2 people pulling a calf to be around 150 kg whereas a calf jack can exert 400 kg of force (30). These reported assisted calving forces are 2–5 times greater than the 75 kg of force estimated to be exerted by a cow calving normally (i.e., without assistance) (30). Becker et al. (25) investigated the peak forces applied to newborn calf cadavers when the legs were pulled, alternating one and then the other vs. pulled simultaneously through a preserved cow pelvis. They reported mean maximum forces applied when the calf's chest entered the pelvis to be between 352 and 547 N (35.9–55.8 kg) pulling on one limb at a time compared to between 554 and 597 N (56.5–60.9 kg) when both limbs were being pulled simultaneously. These forces reported are lower than what is reported in the present study. Lower forces described by Becker et al. (25) may be because the calves used in that study were dairy breeds whose body dimensions are different from beef calves, which has been associated with the degree of calving difficulty (13, 31). The calves were also cadavers, which may impact the force required for delivery due to changes in malleability and elasticity of the tissues compared to a live calf. In contrast, in the present study all calves were assisted at birth due to lack of progression of parturition and alive at birth, depicting real-life scenarios. Thus, the estimates of forces applied to calves assisted at birth in the present study are more reflective of what actually occurs in the field.

An *in vivo* study investigating the use of an antispasmodic drug in cattle to relax the myometrium measured the duration of calving and multiple forces (32). Lange and colleagues (32) used 3 categories defined by the range of total force required to deliver the calf (light = 0–50.9 kg; moderate = 51.0–101.9 kg; heavy > 102.0 kg) to classify the degree of calving difficulty. The maximum pulling force measured was 1979.5 N (201.8 kg) and the average duration was 3.6 minutes. In contrast, the greatest peak force on both legs in the present study was 346.2 kg and the average duration was 1.9 minutes for manual delivery and 5.2 minutes for mechanical delivery. Those authors (32) also measured the total force applied during delivery and found a range from 64,373 N seconds to 91,553 N seconds (109.4 kg min - 155.5 kg min). These total forces were lower than the present study and may be due to the effects of the antispasmodic drug causing relaxation of the uterus and decreased constriction on the fetus. Interestingly, that study noted that calving ease is difficult to standardize and so research projects attempting to objectively classify the degree of calving difficulty could use force parameters as a method of objective classification for calving difficulty.

Trauma has been associated with calving difficulty and increased forces in *in vivo* studies (11, 24). Specifically, in a study conducted by Wehrend and colleagues (24), pulling forces were categorized into light (490 N, 49.9 kg), moderately heavy (784 N, 79.9 kg), and heavy pulling forces (980 N, 100.0 kg), and those authors found traumatic lesions of the birth canal of the dams when moderately heavy and heavy pulling forces were applied. However, the maximum force recorded in that study was 1471 N (149.9 kg) because the model of fetal extractor used could not exert more than 150 kg of force. That study also found an association between increasing forces and increasing durations of extraction (24). In a study quantifying the amount of subclinical trauma in association with calving difficulty, blood biomarkers for muscle damage (creatine kinase and aspartate aminotransferase) were measured in calves 24 h after calving (11). For calves experiencing difficult births, biomarkers of muscle damage were significantly higher than easy assisted births or unassisted births (11). Subclinical trauma was associated with decreased vigor and an increased risk for inadequate transfer of passive immunity (11). These studies indicate that difficult births require increased pulling forces, leading to trauma to both the dam and the calf.

Although it is unknown what threshold of these forces should be considered maximal for the well-being of the calf, the forces found in this study for mechanical deliveries were lower than the range of forces that created femoral fracture (3.7–10.6 kilonewtons; 295–1,091 kg) found in a previous cadaver study (23). In that study, they investigated the amount of force necessary to create femoral fractures of calf femurs in a model simulating the most common location of femoral fractures associated with calving difficulty. In addition to trauma, other factors such as acidemia and hypoxia have been associated with a prolonged calving or increased force of traction during calving assistance (13–15). These factors increase the risk of mortality in neonatal calves (33). It is interesting to note that all the calves (*n* = 3) that died of causes associated with a difficult birth in the present study were mechanically delivered. Therefore, future studies are warranted to investigate the impacts of excessive forces on calf health and survival.

Cow-calf operations differ in the way they manage assisted calvings (20). These differences are reflected in this observational study by differences in forces and birthweight between Ranch A and B. The differences in birthweight between the ranches enrolled may be due to differences in breed of cattle (Angus [Ranch A] vs. commercial, mixed breeds [Ranch B]) and what decision was made by the rancher for the method of delivery (using fetal extractor immediately [Ranch A] vs. trying to manually deliver the calf first then switching to a fetal extractor [Ranch B]). These differences in management decisions are reflective of the variability amongst cow-calf producers in the field. The decision to attempt manual delivery first and then switch to mechanical delivery may have increased the duration of calving assistance and cumulative force but not the peak forces or maximum jerk force on Ranch B. Although multiple ranch personnel delivered calves at both ranches, manually delivered calves did not differ between the two ranches, suggesting that the method of delivery had more of an impact on forces applied to the calf than the person delivering the calf. Although birthweight has been associated with calving difficulty and is considered a good predictor of the degree of calving difficulty (19, 21, 22, 34), it was not significantly different by method of delivery in this study. Bull calves can experience a higher incidence of calving difficulty than heifer calves. This association is influenced by bull calves tending to have greater birthweights than heifer calves, and when birthweight is accounted for as a confounder, the association between sex of the calf and calving difficulty can be reduced (13). The proportion of bull or heifer calves was not significantly different by method of delivery in this study. Therefore, mechanical extraction applied greater forces on the calf regardless of birthweight or calf sex so method of extraction and ranch appear to be more influential on the forces applied to assisted calves.

Although this research has significant implications, such as quantifying the amount of force applied to calves assisted at birth, there were some limitations. Due to on-farm protocols, it was not possible for research personnel to be blinded to calving difficulty during data collection although the automated collection of the force data should have allowed these measurements to retain their objectivity. The duration of assistance of all deliveries in this study were under 10 min, but under the field conditions of this study, it was not feasible to measure exactly how long the dams were in stage 2 of labor before the decision to intervene was made. On both ranches, the policy was to wait one to two hours from the time fetal membranes or feet were observed before intervening; however, the exact moment that stage two labor began was rarely observed. Although multiple ranch personnel assisted calvings at each ranch and could have influenced the method of delivery and forces applied, the same veterinary practice trained both ranches' personnel for appropriate calving intervention strategies. A study by Schuenemann and colleagues (35) demonstrated increased knowledge and application of appropriate calving assistance techniques by farm personnel after formal training. It was observed that common ranch protocol on Ranch A included proactive use of a calf jack, which may not be representative of other beef operations. It is important to note that clinically there is a need to balance the risk of hypoxemia by quickly extracting the calf with the trauma of a forceful delivery. Further research is needed to determine the critically traumatic peak force point to determine when a cesarean section is preferable and to establish guideline for maximal advisable duration of delivery.

## Conclusion

This study confirmed the effectiveness of the CAF-Log system as a method to measure the duration and various force parameters applied to a calf during assistance at birth and established that significantly greater forces were applied to mechanically delivered calves compared to manually delivered calves. The CAF-Log system could be used for future studies to investigate different forces applied to a calf during calving assistance and their impacts on cow and calf health and productivity.

## Data Availability Statement

The raw data supporting the conclusions of this article will be made available by the authors, without undue reservation.

## Ethics Statement

The animal study was reviewed and approved by Canadian Council on Animal Care by the University of Calgary Veterinary Sciences Animal Care Committee (AC15-0150). Written informed consent was obtained from the owners for the participation of their animals in this study.

## Author Contributions

MW was the principle investigator for this project. MW, JP, CT, and AK participated in data collection and analysis. MW, JP, CT, MU, and EP participated in interpretation of the results. All authors participated in study design and preparation of the manuscript.

## Conflict of Interest

The authors declare that the research was conducted in the absence of any commercial or financial relationships that could be construed as a potential conflict of interest.
